# A Rare Case of Torsion of the Intra-abdominal Testis: A Tangle Beneath

**DOI:** 10.7759/cureus.90237

**Published:** 2025-08-16

**Authors:** Ateeq Omer, Gopakumar Govindapillai, Ajay Krishnan

**Affiliations:** 1 Emergency Medicine, Holy Family Hospital, Thodupuzha, IND; 2 General Surgery, Holy Family Hospital, Thodupuzha, IND; 3 Emergency, Holy Family Hospital, Thodupuzha, IND

**Keywords:** absent testis, cryptorchidism, ectopic testis, intra-abdominal testis, torsion testis

## Abstract

Cryptorchidism is the most common congenital anomaly of the male genitalia, and if left untreated, it can cause infertility, inguinal hernias, testicular torsion, and an increased risk of testicular germ cell cancer. Torsion of the intra-abdominal testis is a rare cause of acute abdomen that is often misdiagnosed unless there is a strong index of suspicion, a clear history of cryptorchidism, and a scrotal examination. We present a case of torsion of the intra-abdominal testis diagnosed intraoperatively during surgery performed for suspected appendicitis. We also review the management of cryptorchidism.

## Introduction

The male reproductive glands, known as the testes, have two functions: exocrine (spermatogenesis) and endocrine (testosterone production). Testes start to form at eight weeks of gestation as an intra-abdominal organ and later descend to become extra-abdominal. Cryptorchidism is a condition in which one or both of the testicles fail to descend into the scrotum. Cryptorchidism is the most common congenital anomaly of the male genitalia, and if left untreated, it can cause infertility, inguinal hernias, testicular torsion, and an increased risk of testicular germ cell cancer. The rotation of the testes and epididymis around the spermatic arteries causes testicular torsion, which abruptly stops the flow of blood to the testicles. Testicular torsion is a urological emergency, and the patient's prognosis is contingent upon when they arrive at the emergency department, as well as how promptly it is diagnosed and treated. Delays in diagnosis and treatment always lead to testicular loss. In a normally descended testis, around 20% to 40% of cases of testicular torsion result in an orchiectomy. For those who present within the first 6 hours of symptoms, the salvage rate is nearly 90%, but this number quickly drops to less than 50% if the delay in seeking help is more than 12 to 24 hours [1,2]. However, in the case of undescended testis, usually patients present late or have vague symptoms, leading to delayed or missed diagnosis and salvage rate in less than 20%. Here we present a case of torsion of the intra-abdominal testis diagnosed intraoperatively during surgery performed for suspected appendicitis.

## Case presentation

**Figure 1:**
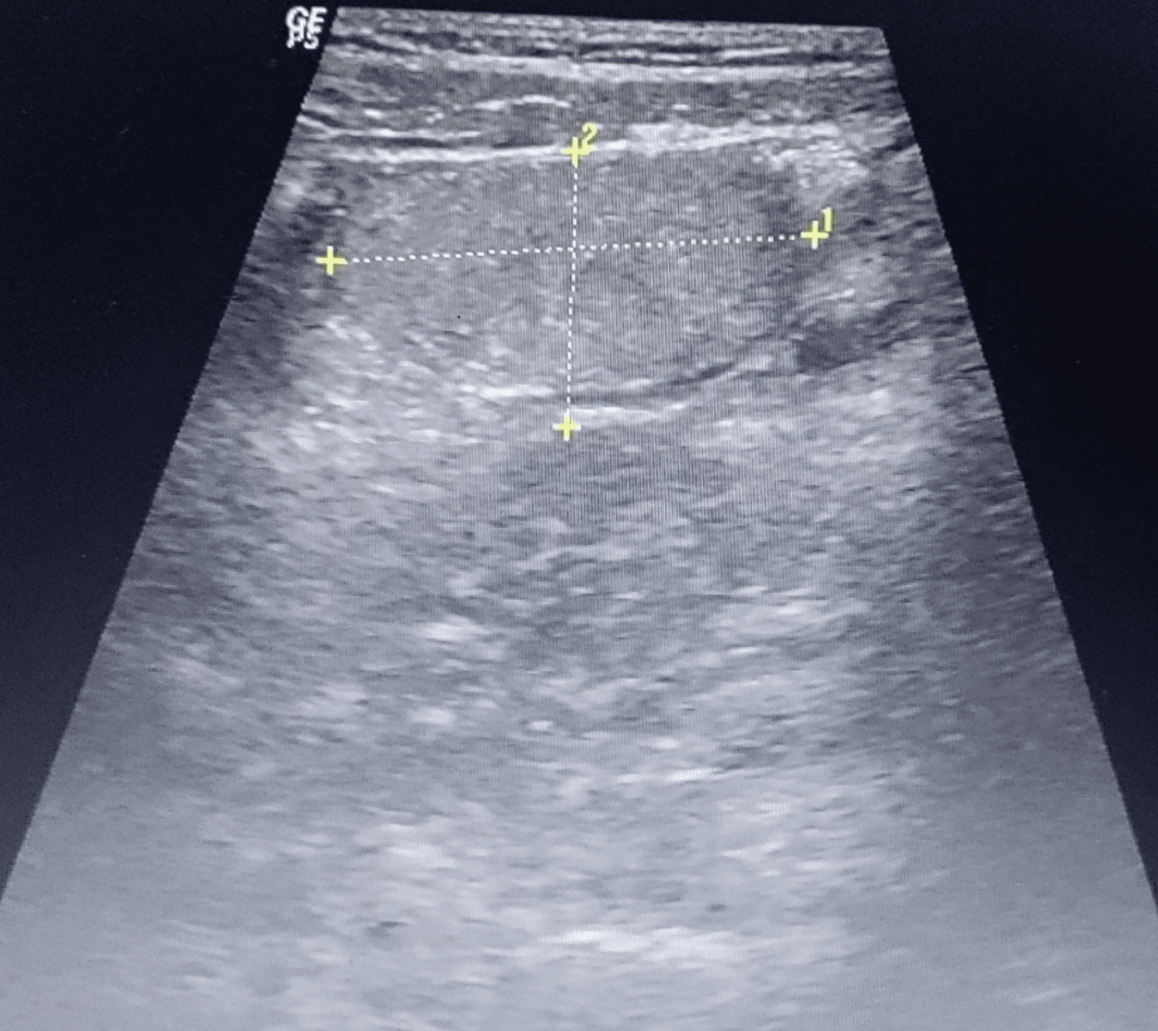
Ultrasound showing an isoechoic area in the right inguinal fossa

A 25-year-old male patient reported to the emergency department with a four-day history of right-sided lower abdomen pain, vomiting, and fever. On examination, the patient had tenderness in the right lower quadrant of the abdomen. The investigation revealed leukocytosis, with a total count of 14,370/cumm (normal range 4,000-10,000/cumm) and CRP (C-reactive protein) of 15.39 mg/L (normal range <6 mg/L). Ultrasound imaging of the abdomen revealed an irregularly shaped isoechoic lesion measuring 2.5x2.0 cm in the right iliac fossa (Figure 1). The appendix could not be visualized.

**Figure 2:**
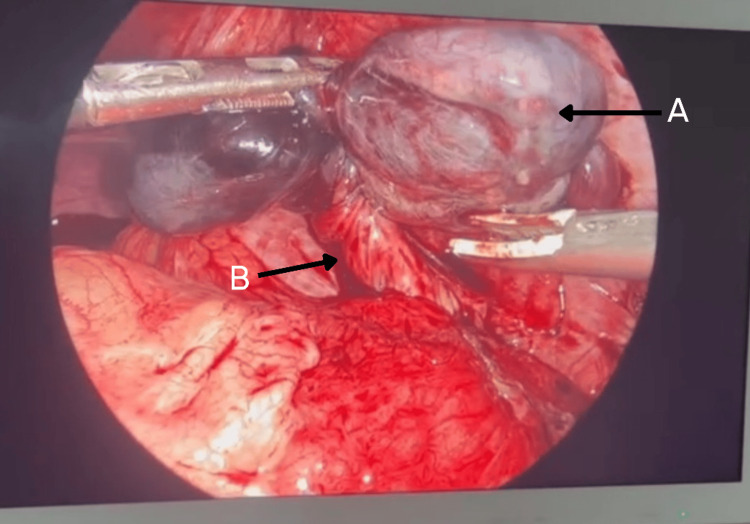
Laparoscopic view of the intra-abdominal testis A. Ischemic testis. B. Twisted spermatic cord.

**Figure 3:**
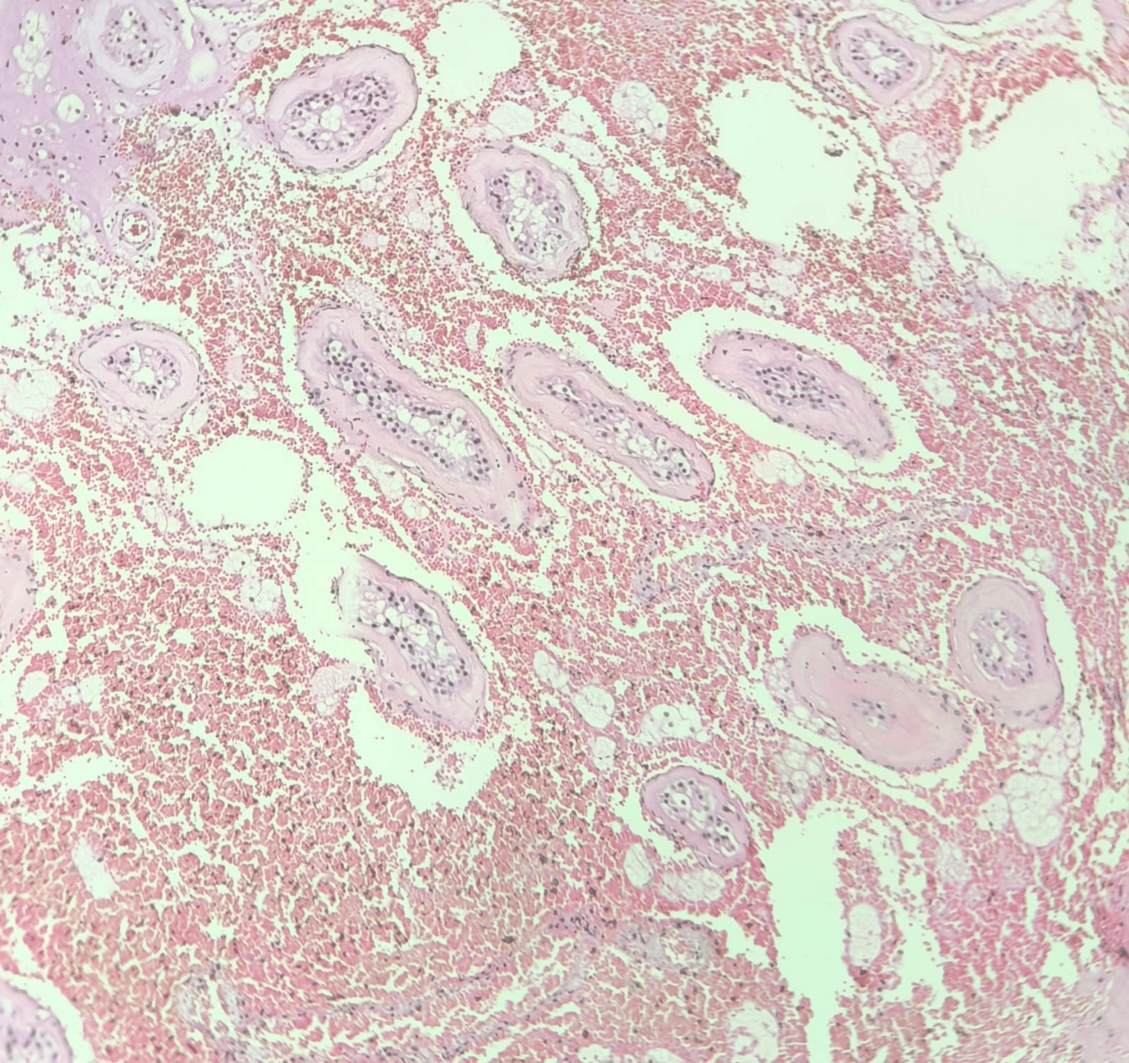
Microscopic view of cryptorchid testis showing atrophic seminiferous tubules with absent spermatogenesis and stroma showing hemorrhage (x10).

**Figure 4:**
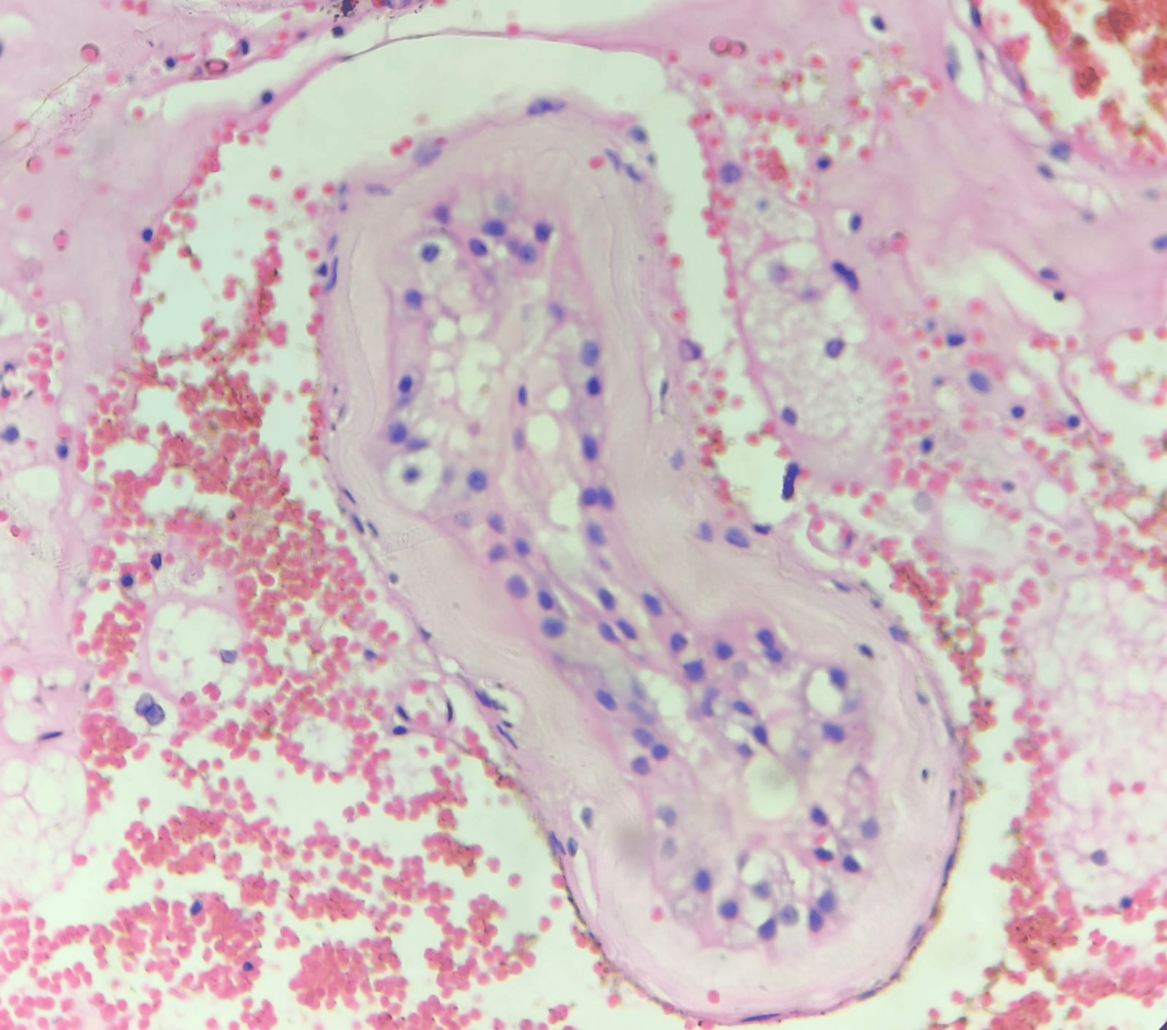
Histology showing atrophic seminiferous tubules (x40)

With a suspicion of acute appendicitis, laparoscopy was performed, which revealed torsion of the intra-abdominal testis (Figure 2), and orchidectomy was also performed. The specimen was sent to the pathologist for histopathological studies. Microscopy revealed seminiferous tubules exhibiting marked atrophy and peritubular fibrosis, consistent with an infarcted and torted cryptorchid testis. No evidence of dysplasia or malignancy was identified. Seminiferous tubules showed decreased to absent spermatogenesis with scattered microliths. Stroma showed dense areas of hemorrhage with necrosis (Figures 3, 4). Later, ultrasound of the scrotum was performed, which showed normally descended left testes and an empty right hemiscrotum. The patient had an uncomplicated recovery and was discharged home the day after the surgery.

## Discussion

Cryptorchidism is the most common congenital abnormality of male genitalia in which one or both testes is not in the scrotum. This could result from an undescended testis or from its absence as a result of agenesis or atrophy due to intrauterine vascular impairment. Testes start to form at eight weeks of gestation as an intra-abdominal organ and later descend to become extra-abdominal. The testes move to the inguinal area during the first stage of testicular descent, which is between 10 and 15 weeks of pregnancy. Between 25 and 35 weeks, the second inguinoscrotal phase takes place [3,4]. A number of factors contribute to normal testicular descent, including the testis's development, the hormones testosterone and insulin-like growth factor 3, the hypothalamic pituitary testicular axis, a patent processus vaginalis, gubernacular outgrowth and regression, and intra-abdominal pressure [5]. Cryptorchidism may result from any deviation in the aforementioned factors, and additional etiological causes include genetic predisposition, environmental variables, and associations with numerous syndromes such as KBG syndrome, Kallmann syndrome, and Noonan syndrome [6].

Around 30% of preterm male infants and 3% of full-term male infants are born with one or both testicles undescended. Around 80% of cryptorchid testes, however, descend within the first three months of infancy [7]. Cryptorchidism can appear unilaterally or bilaterally, with the right testicle showing a higher frequency of involvement. Around 10% of all patients with undescended testicles have bilateral cryptorchidism. If the testis has not descended by six months, spontaneous descent is rare.

The testes become extra-abdominal in the scrotum after development, which helps maintain a temperature lower than the body's average temperature. This is necessary for spermatogenesis, and cryptorchidism consequently impacts fertility. Around 10% to 30% of individuals with unilateral undescended testis may experience infertility, and the risk rises from 35% to 65% or more for those with bilateral disease. If bilateral cryptorchid testes are left untreated, almost 90% of cases will become infertile [8]. Untreated cryptorchidism can also result in psychological problems, inguinal hernias, testicular torsion, and an increased risk of testicular germ cell cancers [9]. According to estimates, testicular torsion occurs 10 times more frequently in undescended testes than in normal scrotal testes. Comparing cryptorchid testes to normally descended testes, the rate of salvage of torsion is lower, possibly as a result of a delayed diagnosis.

Ultrasonography is the modality of choice when evaluating for torsion of the descended scrotal testis. However, with cryptorchidism, ultrasound is not sensitive enough to detect intra-abdominal testes, despite having a sensitivity of 95-97% for inguinal testes. Initial sonographic evaluation may not detect the testes when the torted testis is intra-abdominal; instead, it may only show that the testis is absent within the scrotum and inguinal canal. The best diagnostic and treatment option for boys with a nonpalpable testis is laparoscopy, under the pediatric urology guidelines published by the European Association of Urologists. A significant benefit of the laparoscopic technique, despite its minimally invasive nature, is its visibility and potential to transform diagnostic procedures into therapeutic ones. The sensitivity and specificity of imaging tests are insufficient to change whether exploratory surgery is necessary [10].

Undescended testes are always treated surgically (orchiopexy), and the aim of care is to either remove nonviable testicular remains or put and fix viable undescended testes in a normal scrotal position. It is advised that congenitally undescended testes be surgically treated as soon as possible after four months of age and that the procedure be finished before the child turns two (preferably before one year) [10]. Scrotal placement lowers the risk of blunt trauma injury and torsion (for intracanalicular testes). When done early enough, infertility and testis cancer are possible long-term problems that can be avoided but not prevented by successful scrotal relocation of the testis [11,12]. Patient follow-up and counselling are crucial. Hormonal therapy has been tried prior to surgery in the past, but it has not been shown to be effective in causing testicular descent, and thus it is not advised [13].

Torsion of an intra-abdominal testis is a urological emergency and a rare cause of acute abdomen [14]. It typically presents late and is frequently misdiagnosed, impacting testicular salvage. Without a history of undescended testis and a physical examination of the abdomen, including external genitalia, it is difficult to make a diagnosis and rule out other emergency abdominal diseases without performing a laparotomy. In our case, the main cause of initial misdiagnosis was an incomplete history (the patient did not mention an undescended right testis) and an incomplete physical examination (the scrotum was not inspected during the initial examination).

## Conclusions

Pathology of testicular origin should always be considered, especially in young boys presenting with acute lower abdominal or groin pain. Emergency and primary care physicians must be more aware of undescended testis (cryptorchidism) and its associated complications, particularly testicular torsion. Torsion of an undescended testis often presents atypically with abdominal or inguinal pain rather than scrotal symptoms, which can lead to delayed diagnosis and increase the risk of testicular loss. Thorough evaluation of the external genitalia and inguinal regions should be a routine part of the physical examination in such cases. Improved clinical awareness and routine screening can significantly reduce preventable morbidity and ensure timely intervention.
